# Poly[[bis­[3-(1*H*-tetra­zol-1-yl)propanoic acid-κ*N* ^4^]cadmium]-di-μ-thio­cyanato-κ^2^ *N*:*S*;κ^2^ *S*:*N*]

**DOI:** 10.1107/S1600536812014730

**Published:** 2012-04-13

**Authors:** Jian-Guo Wang, Yuan Zhang, Zhong-Xing Su, Xiang Liu

**Affiliations:** a State Key Laboratory of Applied Organic Chemistry, College of Chemistry and Chemical Engineering, Lanzhou University, Lanzhou 730000, People’s Republic of China; b College of Chemistry and Chemical Engineering, Inner Mongolia University, Hohot 047100, People’s Republic of China

## Abstract

In the title compound, [Cd(NCS)_2_(C_4_H_6_N_4_O_2_)_2_]_*n*_, the Cd^II^ cation is located on an inversion center and is coordinated by two N and two S atoms from four SCN^−^ anions and two N atoms from two 3-(1*H*-tetra­zol-1-yl)propanoic acid (Htzp) ligands in a distorted octa­hedral geometry. The SCN^−^ anions bridge the Cd^II^ cations into a layer structure parallel to (100). A weak intra­molecular C—H⋯N inter­action occurs. The layers are further assembled into a three-dimensional supra­molecular structure *via* classical O—H⋯O hydrogen bonds.

## Related literature

For general background to carboxyl­ate-tetra­zole complexes, see: Yang *et al.* (2009 ▶); He *et al.* (2005 ▶); Yu *et al.* (2008 ▶); Dong *et al.* (2008 ▶); Zhang *et al.* (2009 ▶); Li *et al.* (2008 ▶, 2010 ▶); Xie *et al.* (2010 ▶); Bai *et al.* (2008 ▶); Voitekhovich *et al.* (2010 ▶). 
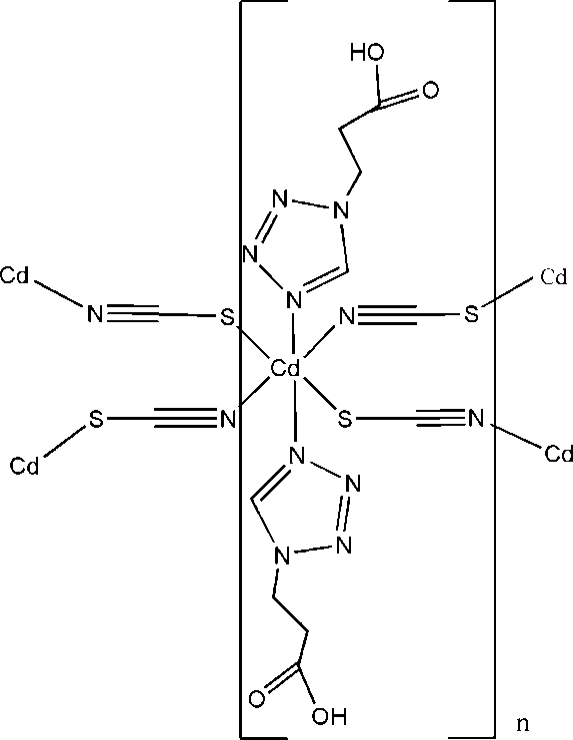


## Experimental

### 

#### Crystal data

[Cd(NCS)_2_(C_4_H_6_N_4_O_2_)_2_]
*M* *_r_* = 512.81
Monoclinic, 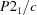

*a* = 12.7402 (19) Å
*b* = 6.9555 (11) Å
*c* = 10.7549 (16) Å
β = 106.809 (1)°
*V* = 912.3 (2) Å^3^
*Z* = 2
Mo *K*α radiation
μ = 1.47 mm^−1^
*T* = 296 K
0.23 × 0.22 × 0.20 mm

#### Data collection

Bruker APEXII CCD diffractometer
Absorption correction: multi-scan (*SADABS*; Bruker, 2001 ▶) *T*_min_ = 0.729, *T*_max_ = 0.758
5775 measured reflections
1695 independent reflections
1505 reflections with *I* > 2σ(*I*)
*R*_int_ = 0.021

#### Refinement

*R*[*F* ^2^ > 2σ(*F* ^2^)] = 0.021
*wR*(*F* ^2^) = 0.052
*S* = 1.07
1695 reflections
128 parameters
H atoms treated by a mixture of independent and constrained refinement
Δρ_max_ = 0.59 e Å^−3^
Δρ_min_ = −0.51 e Å^−3^

### 

Data collection: *APEX2* (Bruker, 2007 ▶); cell refinement: *SAINT* (Bruker, 2007 ▶); data reduction: *SAINT*; program(s) used to solve structure: *SHELXTL* (Sheldrick, 2008 ▶); program(s) used to refine structure: *SHELXTL*; molecular graphics: *SHELXTL*; software used to prepare material for publication: *SHELXTL*.

## Supplementary Material

Crystal structure: contains datablock(s) I, global. DOI: 10.1107/S1600536812014730/xu5497sup1.cif

Structure factors: contains datablock(s) I. DOI: 10.1107/S1600536812014730/xu5497Isup2.hkl

Additional supplementary materials:  crystallographic information; 3D view; checkCIF report

## Figures and Tables

**Table 1:** Hydrogen-bond geometry (Å, °)

*D*—H⋯*A*	*D*—H	H⋯*A*	*D*⋯*A*	*D*—H⋯*A*
O2—H1*O*⋯O1^i^	0.94 (4)	1.70 (4)	2.631 (3)	170 (4)
C1—H1⋯N5^ii^	0.93	2.62	3.404 (3)	142

Symmetry codes: (i) 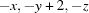; (ii) 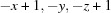.

**Fig. 1.**
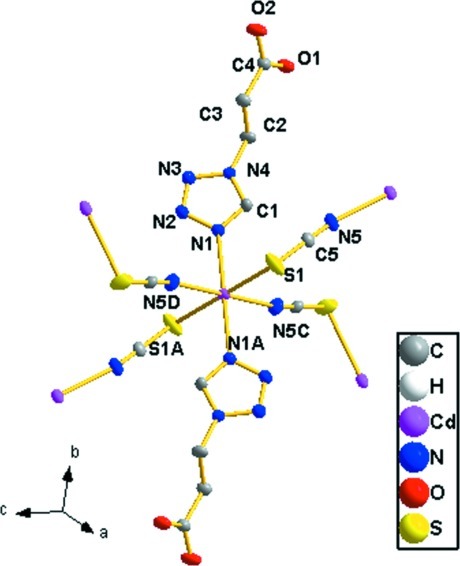
coordination environments of cadmium atoms in the title coordination polymer. Displacement ellipsoids are drawn at the 30% probability level. H atoms have been omitted for clarity.

**Fig. 2.**
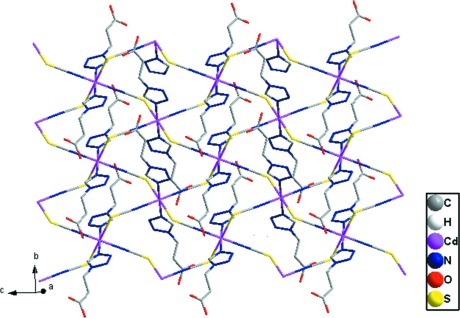
The two-dimensional layer structure of the title coordination polymer along the bc plane. H atoms have been omitted for clarity.

**Fig. 3.**
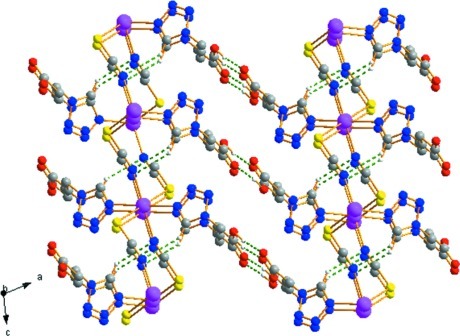
The three-dimensional supramolecular structure of the title coordination polymer. Hydrogen bonds are shown as dashed lines.

## supplementary crystallographic information

### Comment

Recently, the design and synthesis of carboxylate-tetrazole coordination compounds have been of an attractive area of research due to their intriguing topological structures as well as their novel physical properties such as anion exchange, photoluminescence, magnetism behavior and biological activities *etc*. (Yang *et al.*, 2009; Yu *et al.*, 2008; Li *et al.*, 2010; He *et al.*, 2005; Li *et al.*, 2008; Dong *et al.*, 2008; Xie *et al.*, 2010; Bai *et al.*, 2008; Voitekhovich *et al.*, 2010). Herein, we report the structure of the title coordination polymer based on a flexible ligand tetrazole-1-propanoic acid (Htzp).

The title coordination polymer crystallizes in the monoclinic space group *P*2_1_/*c* and the asymmetric unit contains half of the [Cd(Htzp)_2_(SCN)_2_] molecule (Fig. 1). Each Cd^2+^ ion lies on the inversion center of an octahedral environment and is coordinated by two N atoms from two Htzp, two N and two O atoms from four different SCN^-^ ions. Each Cd^2+^ center is linked to four adjacent Cd^2+^centers by four SCN^-^ ions, resulting in a two-dimensional layer structure with Cd···Cd distance of 6.404 Å (Fig. 2). The adjacent two-dimensional layers are further linked through intermolecular hydrogen-bonding interaction between two not coordinated carboxylate group (O2—H1···O1 = 2.631 Å) to afford a three-dimensional supramolecular structure (Fig. 3). In addition, weak intramolecular hydrogen bonds (C1—H1···N5 = 3.404 Å) are present in the crystal structure.

### Experimental

The Htzp (0.0284 g, 0.2 mmol) and NH_4_SCN (0.0152 g, 0.2 mmol) were mixed in distilled water (5 ml) and ethanol (3 ml). Then, CdCl_2_ (0.0367 g, 0.2 mmol) dissolved in distilled water (5 ml) was added slowly to the mixture. The mixture was allowed to slowly concentrate by evaporation at room temperature. Several days later, colorless block crystals suitable for X-ray diffraction were obtained with yield 63% on the basis of Htzp.

### Refinement

Carboxyl H atom was located in a difference Fourier map and refined isotropically. Other H atoms were positioned geometrically and treated in a riding-model approximation, with C—H = 0.93 Å (aromatic) and 0.97 Å (CH_2_) and with *U*_iso_(H) = 1.2*U*_eq_(C).

### Crystal data

**Table:**

[Cd(NCS)_2_(C_4_H_6_N_4_O_2_)_2_]	*F*(000) = 508
*M**_r_* = 512.81	*D*_x_ = 1.863 Mg m^−^^3^
Monoclinic, *P*2_1_/*c*	Mo *K*α radiation, λ = 0.71073 Å
Hall symbol: -P 2ybc	Cell parameters from 4116 reflections
*a* = 12.7402 (19) Å	θ = 3.3–28.3°
*b* = 6.9555 (11) Å	µ = 1.47 mm^−^^1^
*c* = 10.7549 (16) Å	*T* = 296 K
β = 106.809 (1)°	Block, blue
*V* = 912.3 (2) Å^3^	0.23 × 0.22 × 0.20 mm
*Z* = 2	

### Data collection

**Table:**

Bruker APEXII CCD diffractometer	1695 independent reflections
Radiation source: fine-focus sealed tube	1505 reflections with *I* > 2σ(*I*)
Graphite monochromator	*R*_int_ = 0.021
φ and ω scans	θ_max_ = 25.5°, θ_min_ = 3.3°
Absorption correction: multi-scan (*SADABS*; Bruker, 2001)	*h* = −15→15
*T*_min_ = 0.729, *T*_max_ = 0.758	*k* = −8→8
5775 measured reflections	*l* = −13→13

### Refinement

**Table:**

Refinement on *F*^2^	Primary atom site location: structure-invariant direct methods
Least-squares matrix: full	Secondary atom site location: difference Fourier map
*R*[*F*^2^ > 2σ(*F*^2^)] = 0.021	Hydrogen site location: inferred from neighbouring sites
*wR*(*F*^2^) = 0.052	H atoms treated by a mixture of independent and constrained refinement
*S* = 1.07	*w* = 1/[σ^2^(*F*_o_^2^) + (0.0245*P*)^2^ + 0.480*P*] where *P* = (*F*_o_^2^ + 2*F*_c_^2^)/3
1695 reflections	(Δ/σ)_max_ < 0.001
128 parameters	Δρ_max_ = 0.59 e Å^−^^3^
0 restraints	Δρ_min_ = −0.51 e Å^−^^3^

### Special details

**Table:**

Geometry. All e.s.d.'s (except the e.s.d. in the dihedral angle between two l.s. planes) are estimated using the full covariance matrix. The cell e.s.d.'s are taken into account individually in the estimation of e.s.d.'s in distances, angles and torsion angles; correlations between e.s.d.'s in cell parameters are only used when they are defined by crystal symmetry. An approximate (isotropic) treatment of cell e.s.d.'s is used for estimating e.s.d.'s involving l.s. planes.
Refinement. Refinement of *F*^2^ against ALL reflections. The weighted *R*-factor *wR* and goodness of fit *S* are based on *F*^2^, conventional *R*-factors *R* are based on *F*, with *F* set to zero for negative *F*^2^. The threshold expression of *F*^2^ > σ(*F*^2^) is used only for calculating *R*-factors(gt) *etc*. and is not relevant to the choice of reflections for refinement. *R*-factors based on *F*^2^ are statistically about twice as large as those based on *F*, and *R*- factors based on ALL data will be even larger.

### Fractional atomic coordinates and isotropic or equivalent isotropic displacement parameters (Å^2^)

**Table:**

	*x*	*y*	*z*	*U*_iso_*/*U*_eq_	
C1	0.30765 (19)	0.2887 (4)	0.3041 (2)	0.0348 (5)	
H1	0.3447	0.2929	0.2412	0.042*	
C2	0.1466 (2)	0.4991 (3)	0.1939 (2)	0.0370 (6)	
H2A	0.0708	0.4582	0.1737	0.044*	
H2B	0.1698	0.4853	0.1160	0.044*	
C3	0.1555 (2)	0.7069 (3)	0.2350 (2)	0.0342 (5)	
H3A	0.1298	0.7213	0.3110	0.041*	
H3B	0.2318	0.7463	0.2584	0.041*	
C4	0.08935 (19)	0.8341 (3)	0.1283 (2)	0.0314 (5)	
C5	0.42494 (19)	−0.3428 (4)	0.6880 (2)	0.0337 (5)	
Cd1	0.5000	0.0000	0.5000	0.02720 (9)	
N1	0.33986 (16)	0.1944 (3)	0.41393 (17)	0.0345 (5)	
N2	0.26241 (18)	0.2267 (3)	0.4749 (2)	0.0426 (5)	
N3	0.18639 (17)	0.3363 (3)	0.4049 (2)	0.0419 (5)	
N4	0.21474 (15)	0.3766 (3)	0.29673 (17)	0.0289 (4)	
N5	0.46640 (18)	−0.3889 (3)	0.79258 (19)	0.0447 (6)	
O1	0.04537 (15)	0.7745 (3)	0.01892 (15)	0.0402 (4)	
O2	0.08335 (18)	1.0111 (3)	0.16420 (19)	0.0493 (5)	
S1	0.36445 (7)	−0.27644 (12)	0.53866 (6)	0.0594 (2)	
H1O	0.042 (3)	1.083 (6)	0.093 (4)	0.080 (11)*	

### Atomic displacement parameters (Å^2^)

**Table:**

	*U*^11^	*U*^22^	*U*^33^	*U*^12^	*U*^13^	*U*^23^
C1	0.0356 (13)	0.0419 (15)	0.0290 (11)	0.0104 (11)	0.0129 (10)	0.0079 (10)
C2	0.0396 (13)	0.0343 (14)	0.0302 (12)	0.0123 (11)	−0.0007 (10)	0.0047 (10)
C3	0.0360 (13)	0.0327 (14)	0.0299 (11)	0.0036 (11)	0.0032 (10)	0.0038 (10)
C4	0.0322 (12)	0.0298 (14)	0.0303 (11)	0.0015 (10)	0.0061 (10)	0.0033 (10)
C5	0.0348 (12)	0.0343 (14)	0.0324 (13)	−0.0070 (11)	0.0101 (10)	0.0043 (10)
Cd1	0.03311 (14)	0.02849 (15)	0.01786 (12)	0.00767 (10)	0.00396 (9)	0.00081 (9)
N1	0.0377 (11)	0.0369 (12)	0.0300 (9)	0.0113 (9)	0.0113 (8)	0.0079 (9)
N2	0.0506 (13)	0.0425 (13)	0.0407 (11)	0.0153 (11)	0.0227 (10)	0.0164 (10)
N3	0.0435 (12)	0.0452 (14)	0.0424 (12)	0.0132 (10)	0.0209 (10)	0.0129 (10)
N4	0.0304 (10)	0.0281 (11)	0.0271 (9)	0.0063 (8)	0.0064 (8)	0.0041 (8)
N5	0.0533 (13)	0.0522 (15)	0.0279 (11)	−0.0044 (11)	0.0105 (10)	0.0109 (10)
O1	0.0462 (10)	0.0340 (10)	0.0322 (8)	0.0089 (8)	−0.0018 (7)	0.0016 (7)
O2	0.0655 (13)	0.0303 (11)	0.0379 (10)	0.0106 (9)	−0.0076 (9)	−0.0006 (8)
S1	0.0620 (5)	0.0644 (5)	0.0352 (3)	−0.0248 (4)	−0.0121 (3)	0.0207 (3)

### Geometric parameters (Å, º)

**Table:**

C1—N1	1.310 (3)	C5—S1	1.634 (2)
C1—N4	1.314 (3)	Cd1—N5^i^	2.281 (2)
C1—H1	0.9300	Cd1—N5^ii^	2.281 (2)
C2—N4	1.466 (3)	Cd1—N1^iii^	2.3989 (19)
C2—C3	1.506 (3)	Cd1—N1	2.3990 (19)
C2—H2A	0.9700	Cd1—S1^iii^	2.6958 (8)
C2—H2B	0.9700	Cd1—S1	2.6958 (8)
C3—C4	1.500 (3)	N1—N2	1.352 (3)
C3—H3A	0.9700	N2—N3	1.290 (3)
C3—H3B	0.9700	N3—N4	1.343 (3)
C4—O1	1.220 (3)	N5—Cd1^iv^	2.281 (2)
C4—O2	1.299 (3)	O2—H1O	0.94 (4)
C5—N5	1.142 (3)		
			
N1—C1—N4	109.2 (2)	N5^ii^—Cd1—N1	94.85 (7)
N1—C1—H1	125.4	N1^iii^—Cd1—N1	180.0
N4—C1—H1	125.4	N5^i^—Cd1—S1^iii^	92.19 (6)
N4—C2—C3	111.01 (19)	N5^ii^—Cd1—S1^iii^	87.81 (6)
N4—C2—H2A	109.4	N1^iii^—Cd1—S1^iii^	87.15 (5)
C3—C2—H2A	109.4	N1—Cd1—S1^iii^	92.85 (5)
N4—C2—H2B	109.4	N5^i^—Cd1—S1	87.81 (6)
C3—C2—H2B	109.4	N5^ii^—Cd1—S1	92.19 (6)
H2A—C2—H2B	108.0	N1^iii^—Cd1—S1	92.85 (5)
C4—C3—C2	111.32 (19)	N1—Cd1—S1	87.15 (5)
C4—C3—H3A	109.4	S1^iii^—Cd1—S1	180.0
C2—C3—H3A	109.4	C1—N1—N2	105.79 (19)
C4—C3—H3B	109.4	C1—N1—Cd1	129.80 (16)
C2—C3—H3B	109.4	N2—N1—Cd1	124.41 (14)
H3A—C3—H3B	108.0	N3—N2—N1	110.18 (18)
O1—C4—O2	123.9 (2)	N2—N3—N4	106.52 (18)
O1—C4—C3	122.5 (2)	C1—N4—N3	108.31 (18)
O2—C4—C3	113.6 (2)	C1—N4—C2	130.0 (2)
N5—C5—S1	179.4 (2)	N3—N4—C2	121.74 (19)
N5^i^—Cd1—N5^ii^	180.0	C5—N5—Cd1^iv^	164.0 (2)
N5^i^—Cd1—N1^iii^	94.85 (7)	C4—O2—H1O	109 (2)
N5^ii^—Cd1—N1^iii^	85.15 (7)	C5—S1—Cd1	102.24 (9)
N5^i^—Cd1—N1	85.15 (7)		
			
N4—C2—C3—C4	177.9 (2)	Cd1—N1—N2—N3	−179.93 (16)
C2—C3—C4—O1	−7.7 (3)	N1—N2—N3—N4	−0.2 (3)
C2—C3—C4—O2	171.9 (2)	N1—C1—N4—N3	−0.4 (3)
N4—C1—N1—N2	0.2 (3)	N1—C1—N4—C2	180.0 (2)
N4—C1—N1—Cd1	−179.88 (15)	N2—N3—N4—C1	0.3 (3)
N5^i^—Cd1—N1—C1	−40.8 (2)	N2—N3—N4—C2	−180.0 (2)
N5^ii^—Cd1—N1—C1	139.2 (2)	C3—C2—N4—C1	−106.9 (3)
N1^iii^—Cd1—N1—C1	−19 (32)	C3—C2—N4—N3	73.4 (3)
S1^iii^—Cd1—N1—C1	51.2 (2)	S1—C5—N5—Cd1^iv^	23 (27)
S1—Cd1—N1—C1	−128.8 (2)	N5—C5—S1—Cd1	128 (26)
N5^i^—Cd1—N1—N2	139.1 (2)	N5^i^—Cd1—S1—C5	142.55 (11)
N5^ii^—Cd1—N1—N2	−40.9 (2)	N5^ii^—Cd1—S1—C5	−37.45 (11)
N1^iii^—Cd1—N1—N2	161 (32)	N1^iii^—Cd1—S1—C5	47.80 (11)
S1^iii^—Cd1—N1—N2	−128.94 (19)	N1—Cd1—S1—C5	−132.20 (11)
S1—Cd1—N1—N2	51.05 (19)	S1^iii^—Cd1—S1—C5	−57 (10)
C1—N1—N2—N3	0.0 (3)		

Symmetry codes: (i) *x*, −*y*−1/2, *z*−1/2; (ii) −*x*+1, *y*+1/2, −*z*+3/2; (iii) −*x*+1, −*y*, −*z*+1; (iv) −*x*+1, *y*−1/2, −*z*+3/2.

### Hydrogen-bond geometry (Å, º)

**Table:**

*D*—H···*A*	*D*—H	H···*A*	*D*···*A*	*D*—H···*A*
O2—H1*O*···O1^v^	0.94 (4)	1.70 (4)	2.631 (3)	170 (4)
C1—H1···N5^iii^	0.93	2.62	3.404 (3)	142

Symmetry codes: (iii) −*x*+1, −*y*, −*z*+1; (v) −*x*, −*y*+2, −*z*.
